# RETRACTION: Urethral Tissue Reconstruction Using the Acellular Dermal Matrix Patch Modified with Collagen-Binding VEGF in Beagle Urethral Injury Models

**DOI:** 10.1155/bmri/9817873

**Published:** 2025-08-13

**Authors:** BioMed Research International

RETRACTION: Y. Wang, G. Wang, X. Hou, et al., “Urethral Tissue Reconstruction Using the Acellular Dermal Matrix Patch Modified with Collagen-Binding VEGF in Beagle Urethral Injury Models,” *BioMed Research International* 2021 (2021): 5502740, https://doi.org/10.1155/2021/5502740.

The above article, published online on 15 October 2021 in Wiley Online Library (http://wileyonlinelibrary.com), has been retracted by John Wiley & Sons Ltd and the Section Editor of the journal; Kim Bridle.

The retraction has been agreed upon following an investigation into concerns raised by a third party, which identified a major overlap with a previously published article [1]. All listed authors agree to this retraction.
